# Tuberculosis attributed to transmission within healthcare facilities, Botswana—The Kopanyo Study

**DOI:** 10.1017/ice.2021.517

**Published:** 2022-04-06

**Authors:** Jonathan P. Smith, Chawangwa Modongo, Patrick K. Moonan, Mbatshi Dima, Ogopotse Matsiri, Othusitse Fane, Eleanor S. Click, Rosanna Boyd, Alyssa Finlay, Diya Surie, James L. Tobias, Nicola M. Zetola, John E. Oeltmann

**Affiliations:** 1 Department of Health Policy and Management, Yale University, New Haven, Connecticut,; 2 Peraton, Atlanta, Georgia,; 3 Botswana-UPenn Partnership, University of Pennsylvania, Philadelphia, Pennsylvania,; 4 Division of Global HIV and Tuberculosis, Centers for Disease Control and Prevention, Atlanta, Georgia,; 5 Division of Tuberculosis Elimination, Centers for Disease Control and Prevention, Atlanta, Georgia; 6 Division of Pulmonary and Critical Care Medicine, Augusta University, Georgia

**Keywords:** Tuberculosis, transmission, healthcare facilities, nosocomial transmission

## Abstract

**Objective:**

Healthcare facilities are a well-known high-risk environment for transmission of *M. tuberculosis*, the etiologic agent of tuberculosis (TB) disease. However, the link between *M. tuberculosis* transmission in healthcare facilities and its role in the general TB epidemic is unknown. We estimated the proportion of overall TB transmission in the general population attributable to healthcare facilities.

**Methods:**

We combined data from a prospective, population-based molecular epidemiologic study with a universal electronic medical record (EMR) covering all healthcare facilities in Botswana to identify biologically plausible transmission events occurring at the healthcare facility. Patients with *M. tuberculosis* isolates of the same genotype visiting the same facility concurrently were considered an overlapping event. We then used TB diagnosis and treatment data to categorize overlapping events into biologically plausible definitions. We calculated the proportion of overall TB cases in the cohort that could be attributable to healthcare facilities.

**Results:**

In total, 1,881 participants had TB genotypic and EMR data suitable for analysis, resulting in 46,853 clinical encounters at 338 healthcare facilities. We identified 326 unique overlapping events involving 370 individual patients; 91 (5%) had biologic plausibility for transmission occurring at a healthcare facility. A sensitivity analysis estimated that 3%–8% of transmission may be attributable to healthcare facilities.

**Conclusions:**

Although effective interventions are critical in reducing individual risk for healthcare workers and patients at healthcare facilities, our findings suggest that development of targeted interventions aimed at community transmission may have a larger impact in reducing TB.

Tuberculosis (TB) is an airborne infectious disease that remains a significant public health threat, resulting in an estimated 10 million incident cases and 1.4 million deaths each year.^1^ Healthcare facilities have long been recognized as a high-risk environment for TB transmission, with numerous studies consistently documenting a high risk of TB infection and clinical disease among healthcare workers (ie, nurses, doctors, custodians, and other staff) and patients.^2–9^ The increased transmission of TB in healthcare facilities is most alarming in low- and middle-income countries, where health systems often face both an increased burden of TB patients and insufficient means to properly implement infection control and prevention interventions.^2^

Despite the well-characterized increased risk of TB infection and disease attributed to healthcare facilities, their role in the broader population-level TB burden remains poorly understood. Modeling studies of other high incidence settings, such as localized geographic hotspots and non-healthcare high-risk occupations, have investigated the hypotheses that small but high-risk populations may drive community-wide transmission.^10,11^ However, no study has explicitly examined the role of healthcare facilities, and their contribution to community-wide TB burden remains unknown. Understanding the link between healthcare facilities and the broader TB epidemic has important implications for our understanding of transmission dynamics, intervention strategies, and resource allocation.

In this study, we combined genotypic, geospatial, temporal, and other epidemiologic data from a population-based, molecular epidemiologic study with Botswana’s universal, nationalized electronic medical record (EMR) system to estimate the proportion of TB cases in the general TB epidemic attributable to healthcare facilities in a high incidence setting.

## Methods

### Setting

The Kopanyo study (“people gathering together” in Setswana language) is a population based, prospective TB transmission study conducted between August 2012 and March 2016 at TB clinics and directly observed therapy (DOT) centers serving urban and periurban communities in Greater Gaborone and rural communities in Ghanzi province, Botswana. The study procedures are described in detail elsewhere^12^; all patients with TB disease in the catchment area were eligible for enrollment. Briefly, a major goal of the study was to obtain an *M. tuberculosis* isolate from every diagnosed case of TB within the catchment area, because any missed cases in a genotyping study can result in gaps in our understanding of transmission patterns and clusters of isolates with similar genotyping results.^13,14^ Only patients on TB treatment for 14 days or more prior to study screening, incarcerated persons, or those who did not consent were excluded from the study.

### Data ascertainment

Sputum was collected from each participant for mycobacterial culture and drug-susceptibility testing. The first *M. tuberculosis* isolate obtained from each patient was genotyped (Genoscreen, Lille, France) using 24-locus mycobacterial interspersed repetitive units–variable number of tandem repeats (MIRU-VNTR).^15^ Because the mutation rate is low, MIRU-VNTR genotyping of *M. tuberculosis* is often used to establish putative epidemiologic and transmission links between TB cases.^16^ Participants were interviewed using a survey with questions designed to gather information about demographics, location of residence, work locations, and locations where they socialize or frequent. We also collected unique national identification numbers (OMANG numbers), which are required by law for Botswanan citizens and are used for tracking medical and other social information.

The Botswana Ministry of Health maintains a centralized electronic medical record (EMR) system, which contains detailed information for all outpatient and inpatient visits to any public healthcare facility in Botswana and can be accessed from any healthcare facility using the system throughout the country. This system began collecting data on clinical encounters at hospitals in 2004 and was shortly thereafter rolled out to smaller public and private clinics, health posts, and hospitals across the country. EMR entries document the specific location, type (ie, inpatient or outpatient), date of visit, and health coding for each discrete clinical encounter, including dental care, diagnostics, laboratory, pharmacy, radiography, and hospitalization. We abstracted all discrete clinical encounters for all Kopanyo participants in the EMR database that occurred between March 1, 2004, and December 31, 2018. The EMR data were linked to the Kopanyo data set by OMANG number. For participants with missing or incorrect OMANG numbers, name, sex, and date of birth were used to identify the participants’ EMR data.

### Defining overlapping events using EMR and genotypic data

We overlaid the Kopanyo and EMR data to identify any “overlapping events,” defined as 2 or more participants attending the same health facility on the same day (or longer) with the same MIRU-VNTR profile. This intentionally broad definition included both inpatient and outpatient visits and ignored date of TB diagnosis, ward (if inpatient), or TB treatment status. We then considered TB clinical data to further classify these events. We defined each patient’s infectious period independent of clinical encounters. Based on established TB contact-tracing guidelines, the infectious period began 3 months (93 days) before date of diagnosis and extended 14 days after treatment initiation.^17^ The patient was considered infectious at any inpatient or outpatient visit falling within the infectious period. If an inpatient stay overlapped with the beginning of the infectious period, the infectious period was extended further to begin at the date of admission. This definition ensures that patients diagnosed with TB are considered infectious from the initial clinical encounter (from “day 1”), regardless of whether TB diagnosis was the original indication for the encounter.

All overlapping events were then defined as 1 of 4 possible categories (Table 1). “Potential source-to-secondary events” occurred when 1 or more patients were present at the healthcare facility during their infectious period, and at least 1 other participant was subsequently diagnosed with TB after overlapping with this period. This suggests a source patient may have transmitted to the later diagnosed secondary patient(s), who were enrolled at a later date. “Common unknown source events” occurred when 2 or more patients overlapped at the healthcare facility at the same time and were later diagnosed with TB. In this case, there exists a nonzero probability that transmission at the healthcare facility occurred by an unknown source or nonenrolled case. Given the natural history of TB disease, we assumed patients concurrently diagnosed during the same event did not transmit to each other. “Community transmission” was considered when 1 or more cases were diagnosed at the same event and all other cases were diagnosed prior to the event, or all cases were diagnosed concurrently during the event. “All already diagnosed” events occurred when all patients in the overlapping event were diagnosed with TB prior to the event.

### Identifying participants with potential healthcare facility transmission

**Fig. 1.**
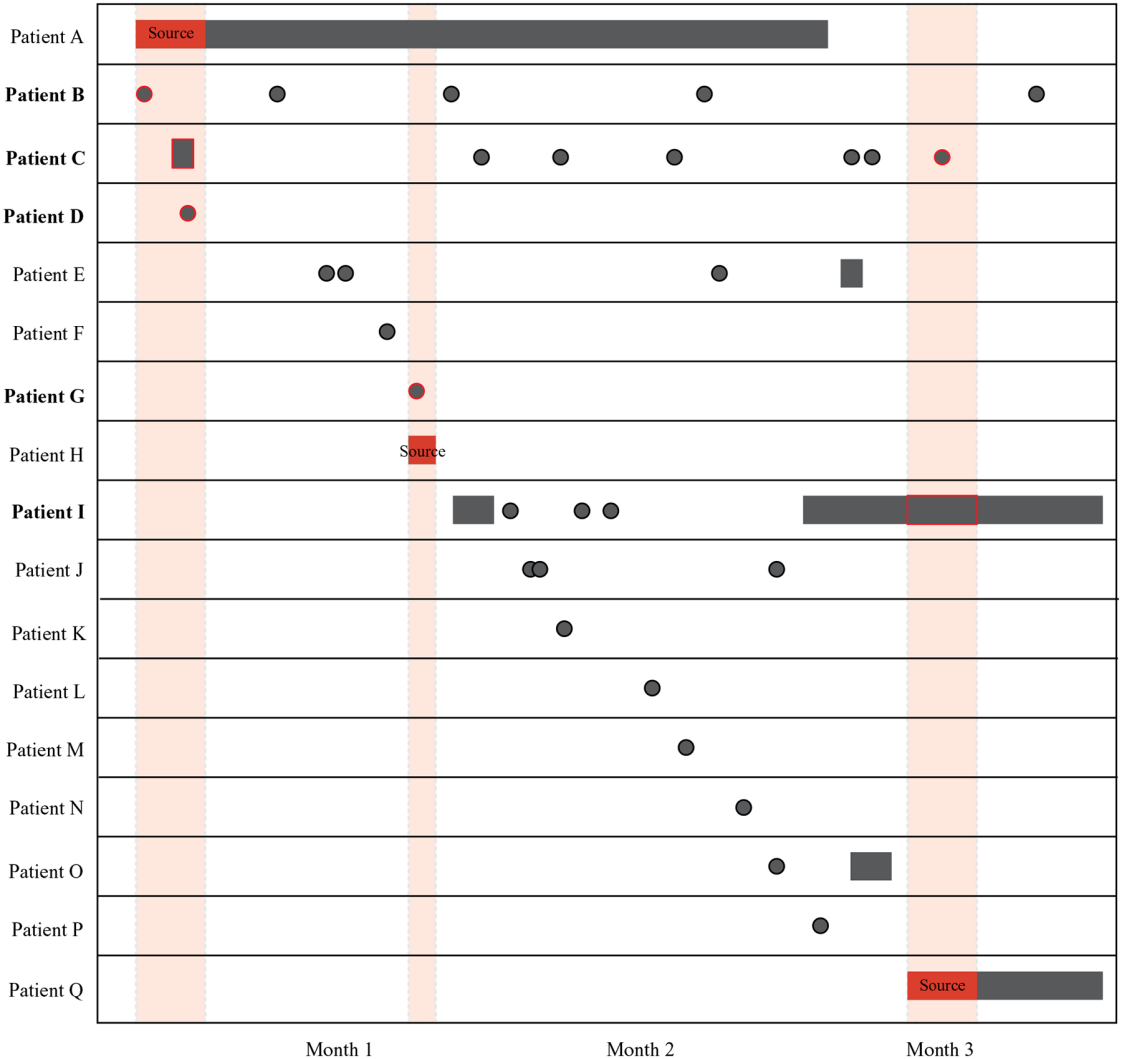
Visualization of source-secondary event. Black indicates either inpatient stays (rectangles) or outpatient visits (circles) at the same healthcare facility. The solid red indicates the infectious period of a potential source case (patients A, H, and Q), as defined in the Methods section. Shaded red areas highlight infectious overlap with potential secondary transmission events (red outline). Participants who potentially became infected at the healthcare facility during the overlapping events are shown in bold (patients B, C, D, G, and I). All patients were diagnosed with TB either during (source cases) or after the overlapping event. Note that this illustration highlights a uniquely complex event for illustrative purposes; the median number of patients in a source-secondary event was 2 (interquartile range, 2–3).

For each unique overlapping event, we identified individual participants within that event who potentially acquired TB at the healthcare facility. First, we excluded all overlapping events defined as “community transmission” and “all already diagnosed,” since transmission at the healthcare facility during these events was not plausible. We included all individuals in “common unknown source” events where at least 1 person developed TB within 2 years of the overlapping event (ie, a 2-year threshold). For participants in a potential source-secondary event, we included any participants who overlapped with the infectious period of a potential source case while at the healthcare facility and developed TB after the event. A potential source case was defined as a participant with an infectious period during the overlapping event while at the healthcare facility. Given the slow progression from infection to active disease, we excluded individuals who were diagnosed with TB concomitantly during the event. Lastly, if 2 or more cases in an overlapping event resided in the same household, we assumed that transmission occurred in the household. An example of a potential source-secondary event is shown in Figure 1.

### Additional sensitivity analyses

Using empirical data in our primary analysis, we estimated the proportion of participants who had potential for transmission at the healthcare facility. According to our definition of an overlapping event, participants were eligible for analysis only if they were successfully assigned to a MIRU-VNTR genotypic cluster. However, the full data set contained participants for whom valid MIRU-VNTR results were not available, yet EMR and clinical data were available. As a secondary analysis we applied a nonparametric resampling scheme that takes maximum advantage of the EMR and clinical data for participants without MIRU-VNTR results.^18,19^ We randomly drew from the distribution of known MIRU-VNTR profiles to assign a MIRU-VNTR result to participants for whom MIRU-VNTR data were missing. We assumed patients assigned a known unique isolate were also considered unique isolates. This resulted in a complete data set with imputed genotype for those with missing MIRU-VNTR, whereas retaining original healthcare facility and clinical data. We repeated this process to create 15,000 unique pseudopopulations, each by resampling with replacement. We then defined overlapping events for each individual pseudopopulation to estimate the number and proportion of cases potentially occurring at a healthcare facility. We calculated the median, interquartile range (IQR), and interdecile range (IDR; between the first and ninth deciles) of these data to estimate the proportion of cases that may be attributable to the healthcare facility.

Additionally, given the considerable variability between infection and clinical TB disease, we expanded our primary 2-year threshold between 1 and 12 years to investigate the impact this assumption has on our overall estimate.

### Ethical approval

This study was approved by the Centers for Disease Control and Prevention Institutional Review Board; the Health Research and Development Committee, Ministry of Health and Wellness, Botswana; and the University of Pennsylvania Institutional Review Boards. Participants provided written informed consent.

## Results

Using data from August 2012 through March 2016, we enrolled 4,331 patients with pulmonary TB in the study. EMR data were obtained for 3,891 (90%) participants; 358 (8%) had no records in the EMR, 80 (2%) had EMR data entered after the end of the Kopanyo study, and 2 (<1%) could not be linked to the EMR database. We detected no statistically significant or clinically relevant differences in culture status, successful assignment of MIRU-VNTR genotype, or age between patients with and without EMR data, though patients with missing EMR records were more likely to be female (57% vs 46%; *P* = .006). We identified 46,853 total clinical encounters at 338 healthcare facilities. Among these, 4,789 (11%) were inpatient visits and 42,064 (89%) were outpatient visits. Across the healthcare system, the median number of inpatient visits per patient was 2 (interquartile range [IQR], 1–3) and the median duration of inpatient stay was 8 days (IQR, 4–16). The median number of outpatient visits per patient was 8 (IQR, 3–14).

Among all enrolled patients, 2,162 (50%) had a positive culture result for *M. tuberculosis*; 1,869 (43%) were clinically diagnosed (ie, diagnosed without culture); and 300 (7%) had culture results for nontuberculosis mycobacteria; 21 (<1%) identified as healthcare workers. Of patients with a positive *M. tuberculosis* culture, 1,924 (89%) had interpretable MIRU-VNTR results, among which 1,881 (98%) had valid EMR data and were included in the analysis. Among included participants, 634 (34%) had unique isolates and the remaining 1,247 (66%) participants shared the same MIRU-VNTR result with at least 1 other participant (ie, TB genotype cluster; Table 2). In total, we identified 236 TB genotype clusters; the median cluster size was 3 (IQR: 2, 4) and the largest cluster size was 137.

In total, 326 unique overlapping events occurred involving 370 individual participants (17%). The median number of participants in each overlapping event was 2 (IQR, 2–3) and the maximum size of an overlapping event was 42 participants. When considering date of TB diagnosis, 38 (12%) events were defined as “potential source-to-secondary” events, 98 (30%) were defined as “common unknown source” events, 95 (29%) were defined as “community transmission” events, and 95 (29%) were defined as “all already diagnosed” events.

Of the 136 overlapping events with the potential for transmission at the healthcare facility, 54 (40%) were excluded in our primary analysis because all patients developed TB >2 years after the event. The remaining 82 overlapping events identified 164 unique participants. No participants in the same overlapping event resided in the same household; 1 (<1%) participant was a healthcare worker. Among these overlapping events, 44 (54%) events were defined as common unknown source events in which biologic plausibility of transmission was assumed for all participants in the group. The remaining 38 (46%) were possible source-to-secondary events, in which each case in the group was individually evaluated for plausibility (Fig. 1).

**Fig. 2.**
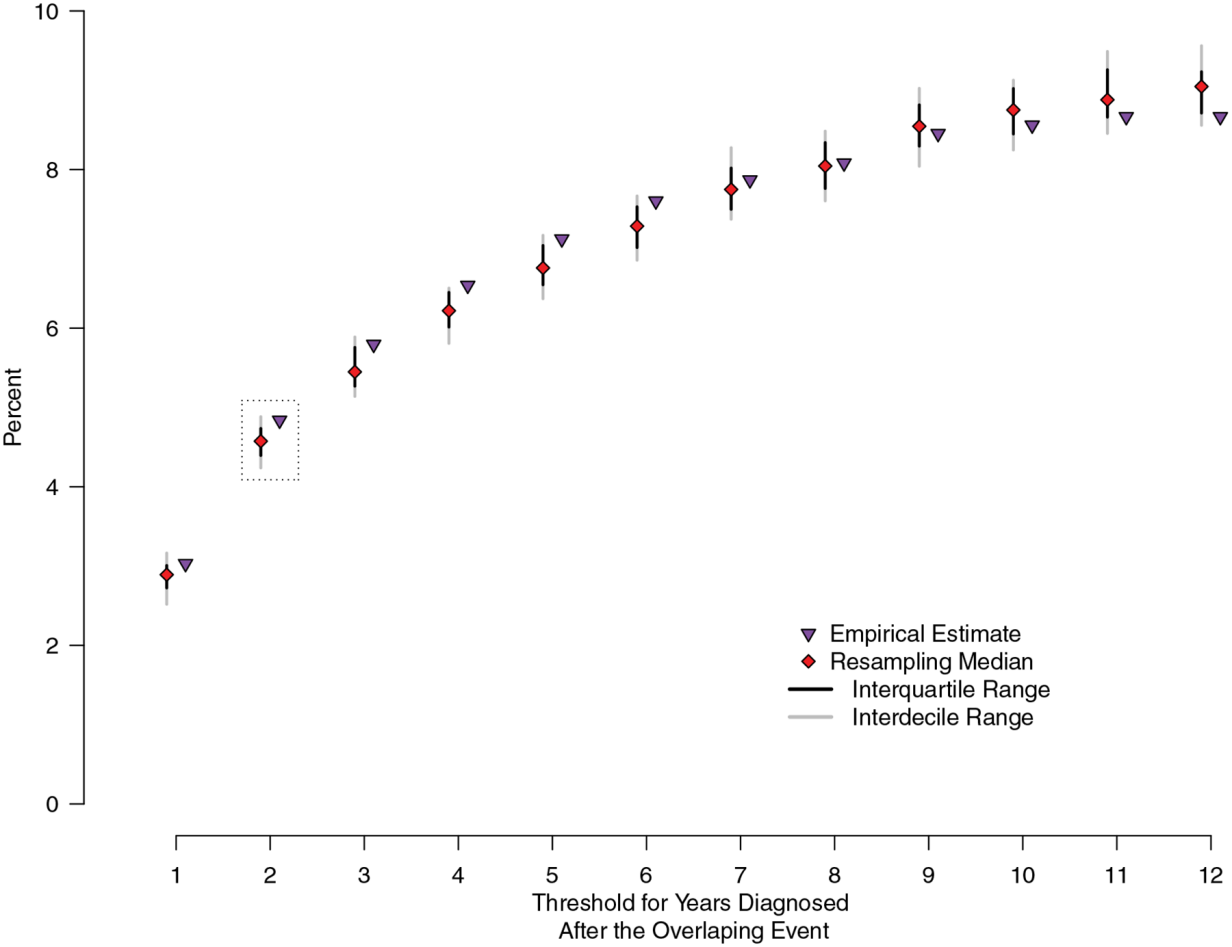
Percentage of transmission potentially occurring at a healthcare facility. The empirical and resampling estimates of the proportion of transmission potentially occurring at the healthcare facility across all possible year thresholds. Our primary analysis considered any overlapping event where a case was later diagnosed within 2 years (grey dotted box). Grey and black lines represent the interdecile and interquartile range, respectively, of 15,000 pseudopopulations resampled according to the methods; red diamonds indicate the median. Purple triangles indicate the empirical estimates using only data with original genetic profiles.

Using a 2-year threshold, 91 (5%) participants potentially contracted TB at a healthcare facility; 47 (52%) of whom were identified from common unknown source events, 36 (40%) from source-to-secondary events, and 8 (9%) from both types of events. The resampling scheme utilizing the full 3,891 participants with EMR data similarly estimated that 5% of participants (median, 178; IQR, 171–184) potentially contracted TB at a healthcare facility. We further evaluated the procedure by restricting the data set to the 1,881 patients with known MIRU-VNTR results, removing the known MIRU-VNTR results, and assigning synthetic MIRU-VNTR results per the methods. The procedure was accurate, with a median of 89 (5%) (IQR, 82 (4%)–98 (5%)) patients identified as potentially contracting TB at a healthcare facility compared to the 91 (5%) in the counterfactual empirical data. Lastly, varying the primary 2-year threshold from 1 to 12 years yielded a range of 3%–9% of potential transmission at a healthcare facility for both empirical and resampled data (Fig. 2).

## Discussion

Using robust EMR data combined with genotypic, clinical, and epidemiological prospective surveillance data, only a small proportion of cases could have plausibly acquired TB infection at a healthcare facility (5%). Despite the known increased relative risk within healthcare facilities these facilities, contribute only a fraction to the overall burden of TB in the community. Thus, while mitigating transmission at the healthcare facilities may have a significant impact on the relative risk among staff, patients, and visitors, our findings suggest targeted community-based interventions may disproportionately reduce TB incidence at the population level.

Our findings are consistent with a limited number of studies that have investigated other similar high-risk groups and their contribution to ongoing TB transmission in high TB-burden settings. A recent study modeling the contribution of high-risk gold mines in southern Africa reported that despite a substantially increased relative risk at the mine, mine workers only account for roughly 4%–9% of incident TB in the community.^10^ Another study modeling public transportation in South Africa reported that annual risk of infection attributable to public transit among daily commuters was 3.5%–5.0%.^20^ Taken together, these data indicate that although high-risk settings contribute to more TB infections on a per-capita basis, most infection is attributable to generalized community transmission. However, these studies are theoretical models not based on molecular epidemiology and thus preclude direct comparisons of results.

Importantly, we quantified this proportion based on biologic plausibility of transmission, not by explicitly defining individual transmission events. Although source-to-secondary events provide a stronger assumption of potential transmission, 52% of participants were identified from common unknown source events. This definition assigns a nonzero probability of transmission to all patients in the event, likely overestimating transmission. Additionally, while wards are often separated by physical air space, most Botswanan hospitals have multiple-occupancy rooms without negative pressure, and many patients are transient during their inpatient or outpatient visit. For these reasons, we defined overlapping events at the broader facility level; we ignored ward-level data because we could not rule out the exposure of shared airspace between patients. We also defined an overlapping event using MIRU-VNTR genotyping, which is less discriminatory than more recent techniques such as whole-genome sequencing (WGS).^21^ Studies with follow up WGS sequencing on MIRU-VNTR clusters have repeatedly demonstrated that MIRU-VNTR clusters often contain multiple sublineages and increased genetic diversity that result in smaller yet more accurate transmission clusters.^22–24^ These intentionally broad approaches seek to ensure our results are conservative and likely an overestimate of the true proportion of transmission occurring at healthcare facilities.

Botswana’s EMR database does not perfectly capture all clinical encounters; the number of individual records that might be missing for linked participants is unknown. Approximately 10% of patients were missing EMR records completely or could not be matched to the EMR database. This is most likely due to patients being seen at small outpatient clinics outside of the EMR system or because data were not entered into the EMR system by participating clinic staff, among other possibilities. Information on relevant overlapping encounters may be lost as a result. A plausible assumption is that missing data in the EMR are correlated with an individual’s propensity to encounter the healthcare system (intuitively, patients with missing data may have fewer encounters with healthcare facilities) and therefore the likelihood of having healthcare transmission is reduced. In this context, our results would likely be an overestimate. However, given that the occurrence of any overlapping event was rare (326 events using the broadest possible definition out of 46,853 clinical encounters), it is unlikely that these missing EMR data would meaningfully change the epidemiological significance of our findings.

Roughly half of the participants were missing MIRU-VNTR genotyping information crucial to our definition of overlapping events. This finding was most commonly a result of culture-negative TB diagnoses (clinical diagnoses), which is typical and this proportion is consistent with other population-based genotypic TB studies in similar settings.^12,25–27^ Missing MIRU-VNTR data in this study did not appear to differ by any demographic or clinical characteristic.^25^ Our resampling procedure to account for these missing data reinforced the empirical results, yet the framework was based on the assumption that genotyping data were missing at random or missing completely at random, assumptions which cannot be verified from the empirical data.

The small proportion attributed to healthcare facilities is likely due to the relatively small proportion of people who spend significant time in healthcare facilities and the generalized nature of the TB epidemic in Botswana. Although a substantial number of low-cost interventions have been developed for the healthcare setting and have shown remarkable results in reducing individual risk (eg, Botswana provides N95 masks to high-risk healthcare workers), our findings suggest that developing targeted interventions aimed at community-wide transmission may have a larger impact in reducing population-level TB incidence in similar high TB burden settings.

## Figures and Tables

**Table 1.** Definitions of Overlap Events

Overlap Event Type	Definition	Biologic Plausibility
Potential source to secondary event	One or more patients were present at the healthcare facility during their infectious period, and at least 1 other participant was subsequently diagnosed with TB after overlapping with this period.	Biologically plausible: A source patient may have transmitted to the later diagnosed secondary patients, who were enrolled at a later date.
Common unknown source	Two or more patients overlapped at the healthcare facility at the same time and were later diagnosed with TB after the overlapping event.	Biologically plausible: There exists a nonzero probability that transmission at the healthcare facility occurred by an unknown source or nonenrolled case.
Community Transmission	One or more cases were diagnosed during the event, and all other cases were diagnosed before the event; OR all cases in the event were diagnosed on the same visit (eg, the event only occurred because all patients presented to the healthcare facility for TB diagnosis).	Not biologically plausible: These scenarios imply transmission occurred in the community, not the healthcare facility.
All already diagnosed	All patients were already diagnosed with TB prior to the overlapping event. TB transmission thus could not have occurred at the healthcare facility.	Not biologically plausible: All cases occurred before the overlapping event.

**Table 2.** Distribution of MIRU-VNTR Cluster Sizes for the KOPANYO Study Participants

MIRU-VNTR Cluster Size	Cluster Frequency	%	Total Cases	%
1 (unique isolates)	634	73	634	34
2	111	13	222	12
3	44	5	132	7
4	24	3	96	5
5	9	1	45	2
6	6	1	36	2
7	8	1	56	3
8	6	1	48	3
9	7	1	63	3
10	1	<1	10	1
11	3	<1	33	2
12	2	<1	24	1
13	2	<1	26	1
14	2	<1	28	1
16	2	<1	32	2
19	1	<1	19	1
21	2	<1	42	2
24	1	<1	24	1
28	1	<1	28	1
29	1	<1	29	2
35	1	<1	35	2
82	1	<1	82	4
137	1	<1	137	7
Totals	870	100	1,881	100

Note. MIRU-VNTR: 24-locus mycobacterial interspersed repetitive units–variable number of tandem repeats.
